# The Role of Ambient Gas and Pressure on the Structuring of Hard Diamond-Like Carbon Films Synthesized by Pulsed Laser Deposition

**DOI:** 10.3390/ma8063284

**Published:** 2015-06-05

**Authors:** Andrei C. Popescu, George E. Stan, Liviu Duta, Cristina Nita, Camelia Popescu, Vasile-Adrian Surdu, Marius-Adrian Husanu, Bogdan Bita, Rudy Ghisleni, Cameliu Himcinschi, Valentin Craciun

**Affiliations:** 1 National Institute for Lasers, Plasma and Radiation Physics, 409 Atomistilor Street, Magurele RO-077125, Romania; E-Mails: liviu.duta@inflpr.ro (L.D.); cristina.nita@inflpr.ro (C.N.); camelia.popescu@inflpr.ro (C.P.); valentin.craciun@inflpr.ro (V.C.); 2 National Institute of Materials Physics, 105bis Atomistilor Street, Magurele RO-077125, Romania; E-Mails: george_stan@infim.ro (G.E.S.); ahusanu@infim.ro (M.-A.H.); 3 Faculty of Applied Chemistry and Materials Science, University Politehnica from Bucharest, 1–7 Gh. Polizu Street, Bucharest RO-011061, Romania; E-Mail: adrian.surdu@live.com; 4 National Institute for Research and Development in Microtechnologies, 126A Erou Iancu Nicolae Street, Voluntari RO-077190, Romania; E-Mail: bogdan.bita@imt.ro; 5 Laboratory of Advanced Materials Processing, EMPA-Swiss Federal Laboratories for Materials Science and Technology, 39 Feuerwerkerstrasse, Thun CH-3602, Switzerland; E-Mail: rudy.ghisleni@empa.ch; 6 Institute of Theoretical Physics, TU Bergakademie Freiberg, Freiberg D-09596, Germany; E-Mail: himcinsc@physik.tu-freiberg.de

**Keywords:** hard carbon thin films, pulsed laser deposition, double stratification, buffer layer, adherence improvement

## Abstract

Hard carbon thin films were synthesized on Si (100) and quartz substrates by the Pulsed Laser Deposition (PLD) technique in vacuum or methane ambient to study their suitability for applications requiring high mechanical resistance. The deposited films’ surface morphology was investigated by scanning electron microscopy, crystalline status by X-ray diffraction, packing and density by X-ray reflectivity, chemical bonding by Raman and X-ray photoelectron spectroscopy, adherence by “pull-out” measurements and mechanical properties by nanoindentation tests. Films synthesized in vacuum were a-C DLC type, while films synthesized in methane were categorized as a-C:H. The majority of PLD films consisted of two layers: one low density layer towards the surface and a higher density layer in contact with the substrate. The deposition gas pressure played a crucial role on films thickness, component layers thickness ratio, structure and mechanical properties. The films were smooth, amorphous and composed of a mixture of sp^3^-sp^2^ carbon, with sp^3^ content ranging between 50% and 90%. The thickness and density of the two constituent layers of a film directly determined its mechanical properties.

## 1. Introduction

Bulk carbon has two crystalline forms: diamond and graphite. In diamond, bonds between carbon atoms are simple (sp^3^ hybridization), whilst in graphite atoms are linked by a double bond, one σ and one π (sp^2^ hybridization) [1]. Under special experimental conditions, amorphous carbon structures with atoms bonded mainly by simple bonds can be obtained. This type of carbon is known in the literature as “diamond-like carbon” (DLC) [2]. Depending on the C-C bonds configuration (sp^3^/sp^2^ ratio) of the DLC films, interesting physical and/or chemical properties are obtained such as high toughness, chemical stability, wear resistance, elastic modulus, biocompatibility and IR transparency [3,4].

The carbon films are usually used in the mechanical and metallurgical industries as protective coatings for cutting blades, magnetic disks, and engine parts, as well as in the glass industry for scratch proof surfaces, and in medicine as protective coatings for implants and prostheses [5]. Carbon films can be found in the literature with the denominations ta-C (tetrahedral amorphous carbon) for films with more than 90% sp^3^ content, a-C (amorphous carbon) for coatings with sp^3^ between 30% and 80% and a-C:H (amorphous carbon—hydrogenated) for samples containing hydrogen and an important part of sp^2^ bonds [6,7].

Numerous deposition techniques have been tested for deposition of DLC layers, the most studied being chemical vapor deposition [8,9,10], pulsed laser deposition (PLD) [11,12,13] and magnetron sputtering [14]. Targets used as raw materials for bombardment or irradiation are usually made of graphite.

A common problem of amorphous carbon thin films is the high internal stress which appears irrespective of the deposition technique, resulting in poor adherence to the substrate and a tendency to peel-off. This drawback can be overcome by a proper selection of the substrate and deposition conditions.

Some articles [15,16] mention the substrate temperature as being critical in the type of structure to be obtained. Temperatures lower than 200 °C enable the synthesis of DLC coatings, whilst temperatures above 200 °C promote the formation of graphite-like coatings.

There have been few reports [17,18] that mentioned particular experimental conditions for which carbon films consisted of two superposed layers which are very different in thickness, composition, and density. We present in premiere a study on the influence of the deposition pressure on the thickness and density of such bi-layer structures for films deposited with the same number of pulses, and how their proportion can influence the overall physical properties of the carbonaceous coating.

## 2. Experimental Section

### 2.1. PLD Experiment

The carbon thin films synthesis was conducted in a stainless steel deposition chamber. A KrF* excimer laser (COMPexPro 205, λ = 248 nm, τ = 25 ns, ν = 10 Hz) (Coherent, Santa Clara, CA, USA) was used for ablation. The laser beam was incident on target at an angle of 45°. The pulse energy was set at 450 mJ and the beam was focused on the target as a rectangular spot with an area of 13 mm^2^. Circular pellets of graphite with diameter of 20 mm and thickness of 3 mm were used as targets. Five thousand consecutive laser pulses were applied to the graphite target in order to obtain one film. Five thousand pulses seemed to be close to the limit for this type of depositions, as the laser entrance window started to have a visible deposition hue when applying more pulses and the laser plasma started to decrease in intensity. Before the actual deposition, a shutter was interposed between the target and the substrate and an irradiation with 200 pulses was conducted to clean the target surface of superficial contaminants. The target was rotated and translated during irradiation to avoid drilling and/or cracking.

The films were deposited on two common substrates used for thin films analysis: Si (100) wafers and quartz. All substrates were cut to an area of 20 × 20 mm^2^. Depositions were conducted at different substrate temperatures, situated in the range of 27–200 °C. Based on adherence tests an optimal deposition temperature of 200 °C was identified, which was kept for all the subsequent deposition experiments. The target-to-substrate separation distance was in all cases 50 mm.

Prior to thin film synthesis, the chamber was evacuated down to a residual pressure of 2 × 10^−7^ mbar. The carbon films were synthesized in vacuum as well as in 0.5, 1, or 10 mbar high purity methane (CH_4_). In order to check for the reproducibility of the results and for statistical analysis, for each deposition condition five samples were synthesized and investigated.

In order to enhance the films adherence to substrate, prior to the PLD deposition, an intermediate buffer layer of SiSi_1−x_C_x_ (0 < x < 1) or SiO_2_(SiO_2_)_1−x_C_x_ was synthesized by co-sputtering using an UVN-75R1 (1.78 MHz) deposition system equipped with two radio frequency magnetron cathodes. The buffer was prepared using two targets (Si (100) or SiO_2_ and graphite) at 3.5 × 10^−3^ mbar argon pressure. The substrate was placed parallel to the Si or SiO_2_ target at a separation distance of 50 mm and was gradually moved during deposition towards the graphite target. After 15 min. of co-sputtering, a layer with a compositional gradient, having a thickness of ~8–10 nm, as estimated from X-ray reflectivity (XRR), was deposited.

### 2.2. Characterization of Deposited Structures

Films morphology was investigated by Scanning Electron Microscopy (SEM) with a FEI Nova Nanosem 630 instrument (FEI, Hillsboro, TX, USA).

Films adherence to substrate was tested by performing pull-out measurements with a Pat Micro AT101 (maximum pull force = 1 kN) (DFD Instruments, Kristiansand, Norway) adhesion tester. The 2.8 mm diameter stainless steel test elements were glued to the film surface with E1100S cyanoacrylate one-component epoxy adhesive. After gluing, the samples were put in an oven for thermal curing (130 °C/1 h). Each test element was pulled-out vertically with a calibrated hydraulic pump until detachment. The experimental procedure was conducted according to the ASTM D4541-09e1 standard. The mechanical adherence strength was determined from the recorded failure value divided by the quantified detached surface area. Five samples were tested independently and mean values and standard deviations were computed.

XRR curves and grazing incidence X-ray diffraction (GIXRD) patterns were acquired using a PANalytical X’Pert PRO MRD instrument (PANalytical, Almelo, the Netherlands) working with Cu*K*_α_ radiation (λ = 1.5418 Å) which was set up in a parallel beam geometry. For the GIXRD measurements the incidence angle was set to 3°, the step size at 0.03° and the time per step at 3 s.

XPS spectra were recorded in a dedicated chamber (Specs GmbH, Berlin, Germany), under ultra-high vacuum (base pressure ~10^−10^ mbar), using Al K_α_ = 1486.71 eV monochromatized radiation. The electrons were collected using a hemispherical electron energy analyzer (Phoibos 150) (Specs GmbH, Berlin, Germany) operated at a 20 eV pass energy. Resolution (in terms of full width at half maximum (FWHM)) of 0.45 eV was achieved. During measurements, a flood gun operating at 1 eV acceleration energy and 1 mA electron current was used in order to ensure sample neutralization. The experimental data were fitted with Voigt profiles *i.e.*, a Gaussian line convoluted with a Lorentzian one. The Gaussian components account mainly for the instrumental resolution while the Lorentzian line is connected to the finite core-hole lifetime associated with the photoionization process. XPS analysis and valence band photoemission, indicating respectively the C1s and O1s core level variation and a transition towards insulating, gapped state which accompanies the sp^3^ bonding of carbon atoms, were used in order to assess the amount of sp^2^ and sp^3^ bonded C in the samples prepared in vacuum and at various CH_4_ pressures.

Raman spectra of thin films were recorded with a Horiba Jobin-Yvon Labram HR 800 spectrometer (Horiba Jobin-Yvon, Longjumeau, France), equipped with a 600 grooves/mm grating, using 442 nm laser radiation. The output power was chosen between 0.2 and 0.4 mW in order to avoid samples heating. For each sample, 30 spectra were acquired with an integration time of 20 s each. The spectra were fitted with Gaussian functions.

A Thermo Scientific Evolution 220 ultraviolet-visible-near infrared (UV-VIS-NIR) spectrophotometer (Thermo Scientific, Waltham, MA, USA) was used to analyze the optical properties of the films in the range 190–1100 nm.

Nanoindentation experiments were carried out using a load-controlled commercial nanoindenter (Hysitron Inc., Eden Prairie, MN, USA). To assess the hardness and reduced elastic modulus of the DLC films, a Berkovich diamond indenter (Hysitron Inc., Eden Prairie, MN, USA) was used for all measurements. We used a single load sequence with constant loading and unloading rates of 20 μN/s, and a holding period of 5 s. A maximum load of 200 μN was applied for all indents which corresponded to indenter contact depths of 15–45 nm, depending on the sample. 15 indentations were conducted for each load and sample. The area function of the tip was determined with the Oliver-Pharr procedure [19].

## 3. Results

### 3.1. Films Adherence to Substrate

Hard coatings are generally brittle and tend to peel-off from the substrate [20]. Initial deposition experiments were conducted with substrates kept at room temperature (RT). All films deposited on Si (100) remained adherent to the substrate, while the thin films deposited on quartz exfoliated, forming flakes either immediately after removal from the deposition chamber, or a few hours after the deposition. In order to increase the films adherence, the substrates were heated. We chose not to exceed 200 °C in order to prevent films oxidation due to chamber degassing at higher temperatures and to keep the vacuum below 10^−7^ mbar during deposition. The films deposited at 100 °C remained adherent irrespective of substrate nature, but those synthesized on quartz were easily exfoliated and produced flakes after a basic mechanical test of scratching with a metallic tip. The most adherent and mechanically resistant films were obtained at a deposition temperature of 200 °C on both types of substrates. They successfully passed the preliminary scotch and scratch mechanical testing. For this reason all further experiments were conducted keeping the substrate temperature at 200 °C.

In the case of films synthesized with a buffer layer, all structures were adherent to the substrate when deposited at 200 °C and no tendency to form flakes was observed.

Next, the films adherence was tested quantitatively by pull-out measurements. The results are summarized in Table 1 as mean values ± standard deviation (n = 5).

**Table 1:** Pull-out tensions needed for detaching carbon films from Si (100) and quartz substrates with or without a buffer layer.

Substrate	Bonding strength [MPa]
SiO_2_	30 ± 0.7
SiO_2_ (buffer)	48 ± 2.6
Si (100)	50 ±9.0
Si (100)/buffer	50 ± 8.0

The bonding strength values of the films decreased by ~40% when using quartz instead of Si (100) as deposition substrate. The (SiO_2_)(SiO_2_)_1−x_C_x_ buffer layer significantly improved the films adherence to the SiO_2_ substrates, seen as an increase of the bonding strength value from 30.5 ± 0.7 to 48 ± 2.6 MPa. The insertion of the co-sputtered transition layer reduced the interface discontinuity between the SiO_2_ and C films, which resulted in an increase of the films adherence to substrate. We note that the SiSi_x_C_1−x_ buffer layer seemed to have no measurable effect upon the films adherence to the Si (100) substrate.

Due to the fact that the growth and adherence of films are dependent on the substrate type, we decided to cancel its influence by synthesizing for the compositional and mechanical analyses only films grown on buffer.

### 3.2. Films Morphology

**Figure 1:**
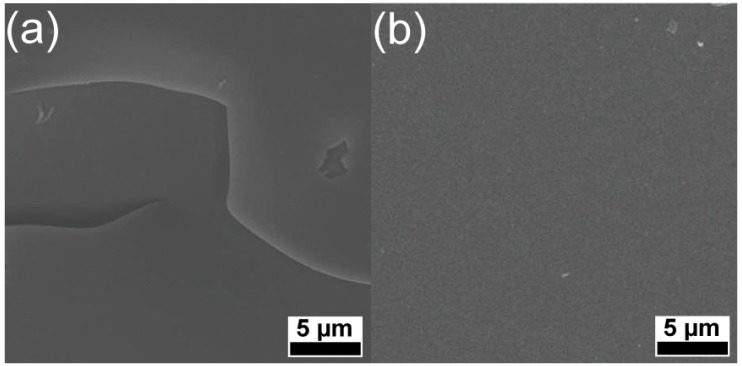
Scanning Electron Microscopy (SEM) micrographs of carbon thin films deposited in vacuum at (**a**) room-temperature and (**b**) 200 °C, on bare quartz substrates.

SEM images at 4000× magnification revealed the general morphology of the PLD carbon films (Figure 1). One can observe in the case of DLC film synthesized on bare quartz at RT (Figure 1a), the presence of exfoliations, in contrast to the compact aspect of the films synthesized onto quartz at 200 °C (Figure 1b).

Irrespective of deposition conditions or the presence or absence of buffer, all films had a very smooth aspect. We mention that there were no differences in films morphology when deposited on bare or buffered quartz or Si (100) substrates.

**Figure 2:**
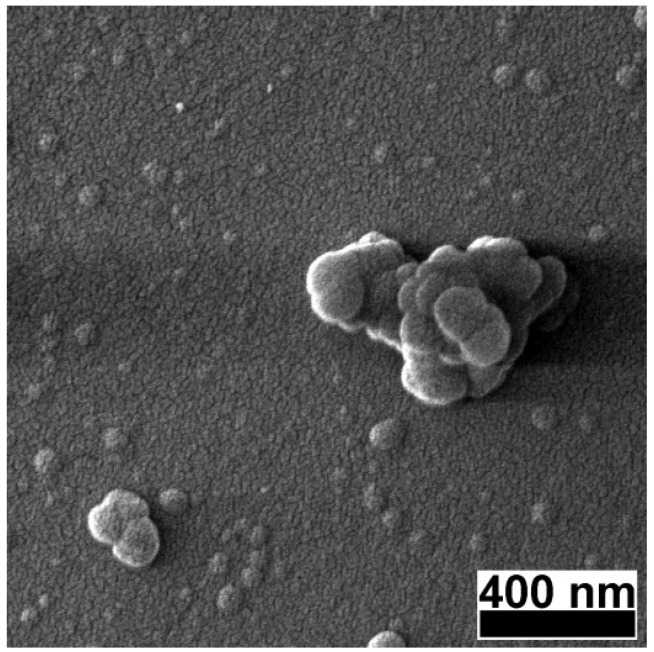
High magnification SEM micrograph of a pulsed laser deposition (PLD) carbon thin film deposited in vacuum on a buffered quartz substrate.

At 120,000× magnification (Figure 2) the films appeared to be built of a uniform bed of nanometric grains over which larger particles of 50–100 nm diameter and particle agglomerates of ~500 nm were deposited. Generically, such type of particles are associated with the molten state of the target surface during ablation, and the most common mechanisms are: liquid phase expulsion due to the blast-wave, hydrodynamic instabilities at the target surface, and subsurface melting [21]. In the particular case of carbon-based targets, liquid phase is improbable, and therefore, the most probable causes for the appearance of droplets are: *i*) explosive dislocation of the substance during plasma plume expansion and *ii*) coalescence of species in plasma plume during the target-to-substrate flight [21,22].

### 3.3. Structure and Composition

Irrespective of the deposition conditions or type of substrate, no sharp diffraction peaks were identified in the GIXRD patterns of the films. The broad peaks centered at 2θ ~ 18° are an indication of their amorphous nature (see Supplemental Materials). This is in accordance with the papers of Capano *et al.* [23] and Huai *et al.* [24] that were the first to show that the transformation of amorphous carbon in graphite occurs at temperatures higher than 200 °C.

**Figure 3:**
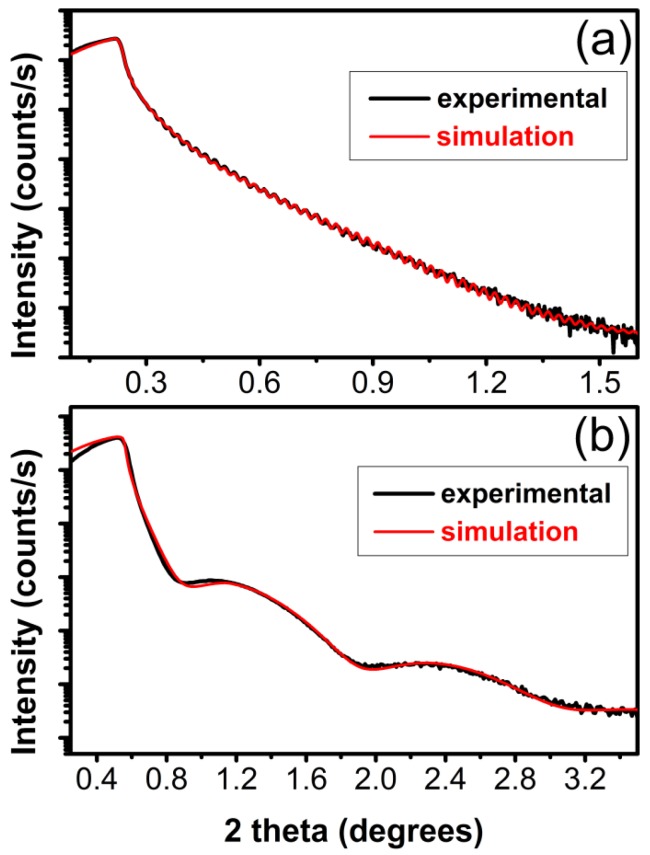
Typical X-ray reflectivity (XRR) patterns (red line) and the best fit (black line) of carbon films obtained in (**a**) vacuum and (**b**) 10 mbar CH_4_ at 200 °C on bare Si (100) substrates.

For the XRR analyses, films were grown on bare Si (100) substrates, in order to avoid the buffer influence on the measurements. Besides the samples synthesized in 10 mbar CH_4_, which were very thin (5–10 nm thick) despite the same number of applied pulses as for the other conditions, most carbon films synthesized by PLD were in fact double layer structures made of carbon with different densities. XRR patterns (black curve) and the best fit (red curve) of films synthesized in vacuum and 10 mbar CH_4_ are given in Figure 3 for comparison.

The top layer had always a lower density compared to the carbon bottom layer. Films synthesized in vacuum had a very thin surface layer of about 30 nm with density of ~1.90 g/cm^3^ and a main bottom layer of ~300 nm or greater with density of 2.4 g/cm^3^. In the case of films synthesized in 0.5 mbar CH_4_, the lower density layer (1.74 g/cm^3^) was dominant with a thickness of ~138 nm, while the bottom layer with a density of 2.51 g/cm^3^ had a thickness of ~95 nm. For a pressure of 1 mbar CH_4_, a low density layer of 1.50 g/cm^3^ and a thickness of ~150 nm was formed on top, while a thicker layer with density of 2.45 g/cm^3^ was present beneath, reaching ~250 nm or even more, since there was no visible feature in the XRR curve coming from the carbon-substrate interface. For the films synthesized in 10 mbar CH_4_ no double stratification was identified and a global density of 2.6 g/cm^3^ was assessed from the simulation.

As a general trend, the bottom layer of the films had densities higher than graphite (2.4–2.6 g/cm^3^ *vs.* 2.267 g/cm^3^), but much lower than diamond (3.515 g/cm^3^).

The information extracted from the XRR patterns is summarized in Table 2.

**Table 2:** Thickness, roughness and density values for the bi- and mono-carbon layers of each diamond-like carbon (DLC) sample, as obtained by XRR studies.

Sample type	Layer	Thickness (nm)	Roughness (nm)	Density (g/cm^3^)
Vacuum	Top	28	0.8	1.9
	Bottom	288	9.7	2.4
0.5 mbar CH_4_	Top	138	1.2	1.7
	Bottom	93	3.8	2.5
1 mbar CH_4_	Top	150	2.0	1.5
	Bottom	245	3.9	2.4
10 mbar CH_4_	Mono	4.3	1.3	2.6

**Figure 4:**
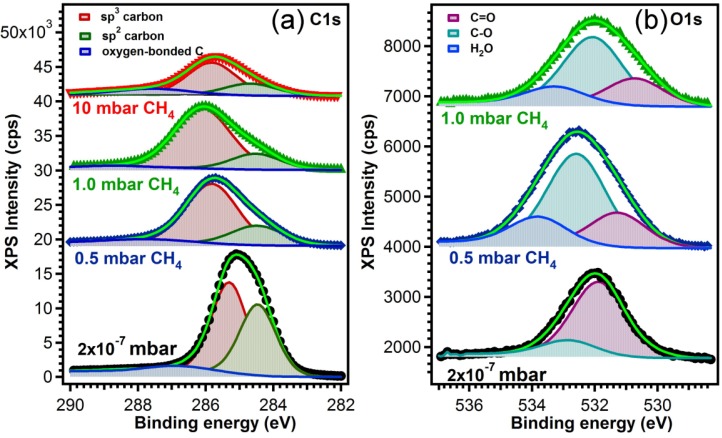
(**a**) C1s core level X-ray photoelectron spectroscopy (XPS) spectra for the samples prepared in vacuum and in different ambient pressures on buffered Si (100) substrates; (**b**) O1s core level XPS spectra for the samples prepared in vacuum and at 0.5 mbar and 1 mbar CH_4_ pressure on buffered Si (100) substrates.

The type of bonds between the carbon atoms in the films synthesized in different atmospheres were assessed by XPS investigations. The high resolution core-level photoelectron spectra of the films are presented in Figure 4. The C1s line of the sp^2^ bond (E = 284.5 eV) was used as reference for the charge shift correction. The component associated to C atoms bonded in the sp^3^ network is shifted to higher binding energies [25,26]. Additional to the main two lines, we used a third one accounting for the contamination caused by the air exposure, and its origin is in the bonding of the surface carbon atoms with the oxygen or water molecules from the air [27,28]. Their shift with respect to the sp^3^ line lies in the range of 1.2–1.6 eV in ether (R-O-R) groups, going to 2.6–2.9 eV for carbonyls (R-C=O) [29] and 4–5 eV for carboxyl groups (-COOH) [30].

The atomic concentrations of sp^2^- and sp^3^-bonded carbon atoms were derived from the analysis of the integral intensities of the corresponding components and all data are summarized in Table 3.

**Table 3:** X-ray photoelectron spectroscopy (XPS) peak separation data for C1s line of DLC films.

Sample type	Vacuum (10^−7^ mbar)			0.5 mbar CH_4_			1 mbar CH_4_			10 mbar CH_4_		
Component assignment	*sp^2^-C*	*sp^3^-C*	O-C*	*sp^2^-C*	*sp^3^-C*	O-C*	*sp^2^-C*	*sp^3^-C*	O-C*	*sp^2^-C*	*sp^3^-C*	O-C*
Component (%)	30.3	55.7	14.0	10.0	86.2	3.8	10.7	88.0	1.3	8.1	61.8	30.1
Position (eV)	284.5	285.3	286.9	284.5	285.8	287.8	284.5	286.1	288.8	284.5	285.8	287.5
FWHM (eV)	1.3	1.45	1.95	1.85	1.95	2.7	1.84	2.1	1.8	1.82	1.95	2.65

Note: O-C*: CO_2_, CO_x_, C=O, C-OH, C-OR, C-OOH.

Its inspection reveals a concentration drop from ~30% sp^2^-bonded C when the deposition was performed in vacuum, to 8% when the deposition was performed in the highest CH_4_ pressure. *A priori*, this change in the sp^2^/sp^3^ ratio should be also reflected in distinct valence band features associated either to materials having a C atom in sp^2^ or in sp^3^ hybridization. Namely, sp^2^ hybridization is known to lead to a finite conductivity in graphitic materials, hence a finite electronic density of states at the Fermi level, and a signature of the π- states located at ~2–3 eV, depending on their localized character [26,31,32,33]. On the other hand, materials with sp^3^ coordination tend to be insulating, with conduction and valence states separated by a gap, which in turn should again be visible in valence band photoemission, accompanied by distinct electronic-σ bands at ~7 eV [34,35].

**Figure 5:**
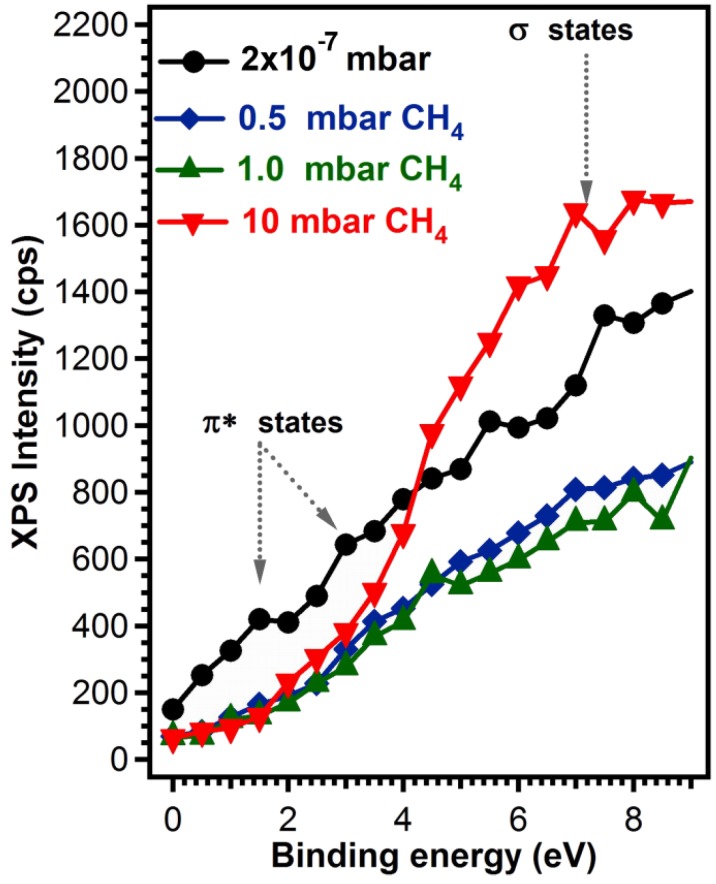
Valence band photoemission for carbon films synthesized in vacuum or in 0.5, 1 and 10 mbar CH_4_ on buffered Si (100) substrates.

We see that indeed this is the case for the spectra recorded in the region near the Fermi level, displayed in Figure 5. The sample prepared in 10^−7^ mbar vacuum conditions, having the highest sp^2^ C-coordinated content presents all these characteristics, and as the sp^2^ concentration drops, the π states intensity also decreases, the system becomes insulating and the σ states, associated to sp^3^ hybridization become more visible.

Concerning the O1s spectra it is worth observing that following the assignment of the various peaks from Ref. [28], it appears that the main contaminants in the case of the system with highest sp^2^ content are the ether groups while for the rest of the samples, the ratio between ether and carbonyl is just the opposite. This is in good agreement with the shift of the third component in the C1s spectra which accounts for oxygen-bonded carbon, which, as can be seen from Table 3 shifts towards higher binding energies as should be in the case of carbonyl groups. The fact that the third component in the O1s spectra, accounting for H_2_O contamination is absent in the sample prepared in vacuum and having the highest sp^2^ content, has to be linked with the hydrophobic character of graphitic materials [36,37].

As an additional observation, we mention that the increase of the FWHM of all lines as the CH_4_ pressure increased, could result from an increase of the atoms arrangement disorder [26].

The sp^2^ carbon content (as extracted from the fitting of C1s peaks) was the highest in the case of depositions conducted in vacuum, reaching a value of ~30% (see Table 3). By increasing the dynamic pressure of CH_4_ during experiments, the sp^2^ content dropped to 10% for 0.5 and 1 mbar CH_4_, and to 8% for experiments performed in 10 mbar CH_4_. The highest sp^3^ content was reached in the CH_4_ pressure interval 0.5–1 mbar. The sp^3^ carbon content increased with the CH_4_ concentration up to ~90% for depositions conducted in 1 mbar CH_4_. Between 1 and 10 mbar the sp^3^ content started to decrease, reaching a value of ~60% for experiments performed in 10 mbar CH_4_.

The C-O fraction for films synthesized in vacuum was ~14%. Films synthesized in 0.5 and 1 mbar CH_4_, had a low amount of C-O bonds of 3.7% and 1.3%, respectively. The increase of the CH_4_ pressure for thin films synthesis to 10 mbar had as effect an important increase in C-O bonding fraction, reaching a value of ~30%.

**Figure 6:**
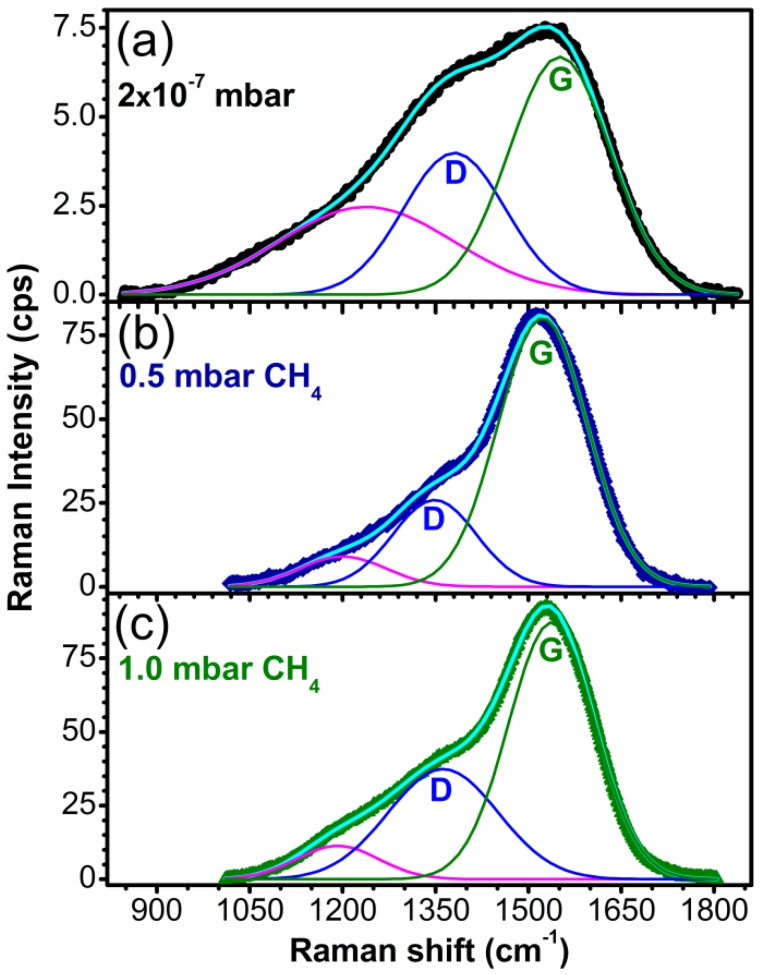
Raman spectra of carbon films synthesized by PLD in (**a**) vacuum; (**b**) 0.5 mbar CH_4_ pressure and (**c**) 1 mbar CH_4_ pressure on buffered Si (100) substrates.

Complementary to XPS analysis which is strictly a surface analysis technique, Raman spectroscopy was also used to characterize the DLC films. The spectra deconvolution of the recorded spectra are presented in Figure 6. The spectra show a distinctive broad band between 1800 and 900 cm^−1^. This band is associated in literature to the overlap of two bands characteristic of amorphous carbon films: G band, representative for C-C stretch in double bonds, which is centered at 1580 cm^−1^ and D band, typical for the breathing mode of the graphitic hexagonal cells, which should appear around 1350 cm^−1^ [38,39,40]. The decrease of the I_D_/I_G_ ratio, the shifting of the G peak towards lower wave numbers and the sharpening of the D-peak are indicators for the decrease of the graphite-like phase concentration in the carbon films [41]. The third component, located between 1300 and 1100 cm^−1^, can be ascribed to nanocrystalline or “amorphous” diamond [42,43,44,45].

The values for the I_D_/I_G_ ratio and the sp^3^ or sp^2^ content were assessed from the sp^3^/sp^2^ dependency on the I_D_/I_G_ ratio and the frequency of the G band given in the works of Ferrari *et al.* [41] and Irmer *et al.* [46].

In the case of the sample synthesized in vacuum, the G band position was set to 1570 cm^−1^ corresponding to an I_D_/I_G_ ratio of 2.6 and a sp^3^ content of 20%–25%. For the sample synthesized in 0.5 mbar CH_4_, the position of the G band was found at 1523 cm^−1^, the I_D_/I_G_ ratio was estimated at ~0.3, with the sp^3^ concentration reaching its maximum (60%–65%). Increasing the pressure to 1 mbar CH_4_ during depositions, the G peak shifted to 1538 cm^−1^ resulting in films with an I_D_/I_G_ ratio of ~0.54 and 50%–55% sp^3^.

The sample synthesized in 10 mbar CH_4_ showed no Raman response, probably because it was very thin. The presence of a strong signal of Si substrate in the spectrum was an additional indication of the very small thickness of this film, being also in accordance with the XPS spectrum which showed the lowest signal for the 10 mbar CH_4_ sample and the XRR curves which indicated a layer with nanometer thickness.

### 3.4. Optical Properties

The Raman and XPS results were corroborated by UV-VIS-NIR spectroscopy results, which also offered information about the degree of clusterization, disorder; and conductivity of the films (the optical band gap being in relation to the sp^2^-sp^3^ content).

**Figure 7:**
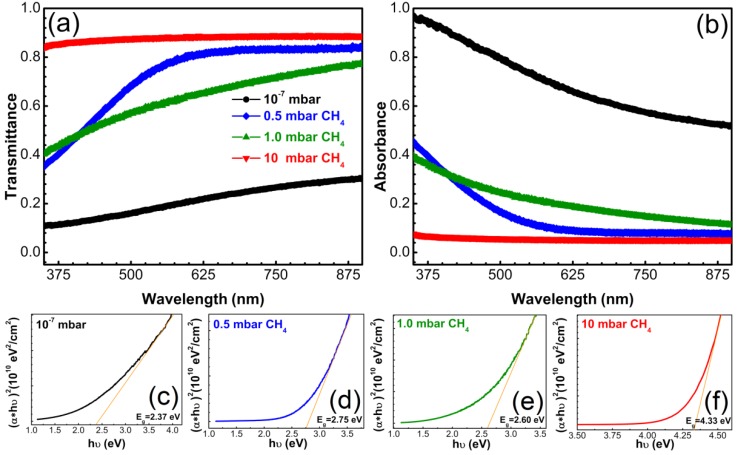
(**a**) Transmittance and (**b**) absorbance spectra of carbon films synthesized by PLD in different deposition ambients on bare quartz substrates; (**c**–**f**) Tauc plots for assessment of the optical band gap.

Figure 7 shows the transmittance and absorbance spectra of the films synthesized by PLD on quartz substrates. No buffer layer was interposed between film and substrate, so as not to influence the optical measurements. The film synthesized in 10 mbar CH_4_ being very thin (~4 nm) had the highest transmittance values of 88% in the 300–900 nm wavelength domain. The films synthesized in 0.5 and 1 mbar CH_4_ ambient showed a poor transmittance in UV up to 350 nm, reaching values of ~36% and 40%, respectively. Transmittance values increased with the augmentation of the incident wavelength, reaching for both films a value of ~80% at 900 nm. However, the trend of the transmittance curves was slightly different: transmittance of films synthesized in 0.5 mbar CH_4_ continuously increased to a maximum of 84% up to ~600 nm and then kept this constant value up to 900 nm. On the other hand, transmittance of films deposited in 1 mbar CH_4_ increased continuously with increase of wavelength. Films synthesized in vacuum had the lowest transmittance which slightly but constantly increased towards higher wavelengths, reaching a maximum of 30% at around 900 nm. This result was anticipated, as these films contained an important sp^2^ component (double bonds) with small band gap between the valence and conduction bands. Thus, the material strongly absorbs light across the whole visible spectrum.

The optical parameters of the DLC films such as absorption coefficient (α), extinction coefficient (k) and band gap (E_g_) were calculated.

The method used for determination of E_g_ was the Tauc plot method. The following expression was used [47]:

(1) $${\left[\left(h\nu \alpha \right)\right]}^{2}=A\left(h\nu -E_{g}\right)$$

where *h*: Planck’s constant; *ν*: frequency of vibration; α: absorption coefficient; *A*: Tauc gap constant and *E_g_* the energy difference between the band gap. The incident photons energy *hν* (eV) was calculated as a function of the wavelength *λ* (nm) according to the relation:

(2) $$h\nu =\frac{1239.7}{\lambda }$$

The absorption coefficient was assessed according to the Beer-Lambert law:

(3) $$\alpha =\left(\frac{1}{d}\right)\left[\text{log}\left(\frac{1}{T}\right)\right]$$

where *d*: path length crossed by the incident light (film thickness) measured in cm and *T*: transmittance. Film thickness was determined from the XRR patterns (Table 2).

The dependency (*αhν*)^2^ on hν was plotted and the linear component of the resulting curves was extrapolated to the *hν* axis. The intercept value was the band gap *E_g_* (Figure 7b–e) [48].

The extinction coefficient “k”, *i.e.*, the decrease of transmitted light intensity due to scattering and absorption, was calculated from Equation (4):

(4) $$k=\frac{\alpha \lambda }{4\pi }$$

where *α*: absorption coefficient and λ: incident light wavelength.

The absorption coefficient and extinction coefficient determine the depth into a material that light can reach before complete absorption. Extrapolating, they are a measure of the extent to which the material absorbs energy. The lower these coefficients, the poorer is the light absorbance, the higher is the band gap and *vice versa* [47].

Most films had semiconductor behavior, with E_g_ of 2.37 eV for layers synthesized in vacuum, 2.75 eV for films deposited in 0.5 mbar CH_4_ and 2.60 eV when increasing the CH_4_ pressure to 1 mbar in the deposition ambient. Films synthesized in 10 mbar CH_4_ pressure were insulators with a band gap of 4.33 eV. The decrease of the E_g_ values is associated in the literature to the increase of sp^2^ content in the films [49]. This is the reason that films synthesized in 0.5 and 1 mbar CH_4_, with the highest sp^3^ content (86% and 88%), had accordingly higher and similar E_g_ values, while films synthesized in vacuum, with 30% less sp^3^ content, had lower values of E_g_. Films synthesized in 10 mbar CH_4_ seemed to be insulators. They exhibited high transparency in accordance with the high values of the band gap. Larson *et al.* [50] showed that the thickness of ~4 nm is a sort of threshold, films up to this thickness being insulators, while thicker films manifest a linear increase of conductance with thickness [51].

**Figure 8:**
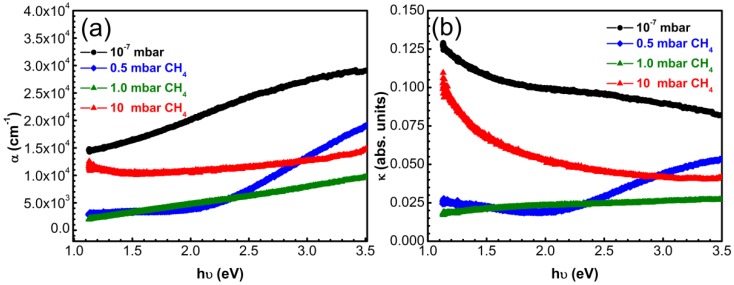
(**a**) Absorption coefficient *α* and (**b**) extinction coefficient *k* *vs* incident photons energy in case of carbon thin films synthesized by PLD in vacuum or CH_4_ on bare quartz substrates.

In the case of the other three type of films, α increases with increasing energy values. The highest coefficients of absorption were exhibited by the films synthesized in vacuum which contain the highest amount of sp^2^-C.

The carbon films fabricated in vacuum displayed extinction coefficients with a descending trend with increase of photon energy. Samples synthesized in 0.5 mbar CH_4_ showed a steeper increase of *k* from 0.02 to 0.05 in the energy range 2–3.5 eV. Samples synthesized in 1 mbar CH_4_ displayed a slow monotonous increase from 0.02 to 0.027 eV.

### 3.5. Mechanical Properties

**Figure 9:**
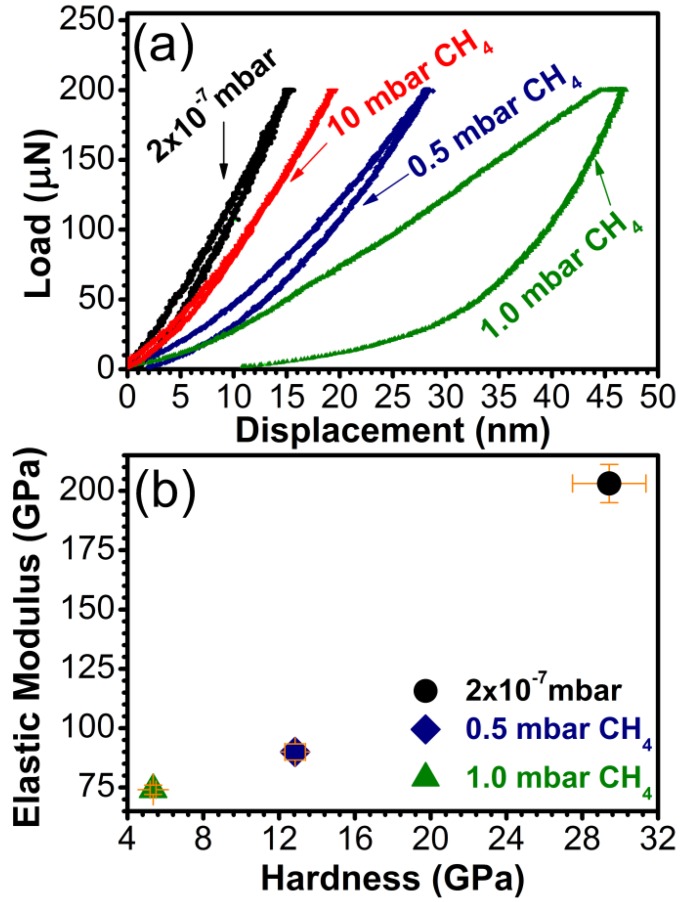
(**a**) Characteristic load *vs.* displacement curves and (**b**) Hardness and elastic modulus for carbon samples synthesized by PLD in vacuum and in 0.5, 1, or 10 mbar CH_4_ pressure on buffered Si (100) substrates.

Due to the hundred nanometers range thickness, the mechanical behavior of the carbon samples was assessed by nanoindentation, which allows the determination of the mechanical properties of thin coatings by penetrating less than 10% of their thickness in the range between 15 and 45 nm. The load/displacement curves are presented in Figure 9a. The hardness and the elastic modulus are given in Figure 9b.

The films synthesized in 10 mbar CH_4_ were too thin (max. 10 nm) for nanoindentation and results were influenced by the substrate which responded elastically to the applied load (see the superposed load and displacement in Figure 9a). The highest values of hardness and elastic modulus were recorded for the samples synthesized in vacuum, *i.e.*, those with the lowest sp^3^ content (~55%). The hardness values for these samples reached ~30 GPa, which classifies them as hard materials [52]. The samples with sp^3^ content over 80% and with a C-O content of less than 5% had a modest mechanical behavior, with a hardness of 5 and 13 GPa, respectively. The films synthesized in vacuum were also the most elastic, with an elastic modulus of 125 GPa, respectively, compared to high sp^3^ content, which had elastic modulus values of under 100 GPa.

For applications requiring high resistance to friction, a lower H or E value is not necessarily a disadvantage for the carbon coating. What should be actually taken into account for predicting the mechanical behavior of a material is the ratio H^3^/E^2^ [53,54]. This term sets the amount of elasticity exhibited by the film [55]. High values of H^3^/E^2^ indicate a highly elastic behavior of the film. In our case the films synthesized in vacuum showed a H^3^/E^2^ ratio of 0.675 GPa, indicative of a largely elastic behavior. The films deposited in 0.5 mbar CH_4_ and 1 mbar CH_4_ indicated a plastic behavior, with values of H^3^/E^2^ of 0.34 and 0.022 GPa, respectively.

## 4. Discussion

The films synthesized in ambient containing CH_4_ are usually thicker than films obtained in vacuum. However, as the CH_4_ amount in the deposition chamber increases, the films thickness decreases. As a relevant example, an increase of the CH_4_ content from 1 to 10 mbar, causes a film thickness reduction by ~95%. The explanation for the films thickness variation is that in vacuum, the ablated matter is not confined and plasma expands divergently in a cone shape. Without a gaseous environment that can reduce the energy of the species, the ablated matter reaches the substrate and literally bombards it. As the gas pressure increases in the reaction chamber, the species in plasma collide with gas molecules from the environment, lose part of their kinetic energy and are confined in a plume following the direction orthogonal to the target surface. If the CH_4_ content is increased too much in the deposition chamber (as it seems for a dynamic ambient pressure of 10 mbar), the ablated molecules can collide multiple times with the gas molecules, thus losing most of their kinetic energy, which is no longer sufficient to ensure their transport up to the deposition substrate.

The films adherence was strongly influenced by the substrate nature. The films were exfoliating from the SiO_2_ substrates, but were adherent when deposited on Si (100). The coefficients of thermal expansion (CTE) between films and substrates seem to be the deciding factor for the film-substrate compatibility. At 25 °C, the carbon films have a CTE of ~2.3 × 10^−6^/°C [56], close to that of pure Si (CTE~3 × 10^−6^/°C) [57]. In the case of quartz, the CTE has a value of ~0.7 × 10^−6^/°C [58]. When the carbon films were deposited on a buffer layer for increasing the compatibility with the substrate, no improvements were observed in the case of the Si (100) substrate. The CTE matching already contributed to a good compatibility between the film and substrate and the graded buffer, made of the same elements with close CTEs, just preserved the good adherence. Because of the markedly CTE mismatch between the carbon films and quartz substrate the films had a tendency to exfoliate and to form flakes. This phenomenon could occur immediately after deposition or after days or weeks, depending on the deposition temperature. The graded buffer layer greatly improved the films adherence to mismatching CTE substrates, the bonding strength increasing by ~60%.

Even though the sp^3^ content quantitative assessment by XPS and Raman spectroscopy differed in terms of output values (due to the different sampling volume—nm range for XPS and whole film thickness for Raman), the evolution trend was the same in the films synthesized under different pressures. The samples synthesized in 0.5 or 1 mbar CH_4_ had the highest sp^3^ content. However, these samples had poor mechanical properties. A possible explanation might be a high content of hydrogen in the samples. It was reported that a high content of hydrogen in the films can be associated with the existence of polymer-like chains of carbon atoms, which weakens the films structure and reduces their mechanical properties [59]. Indeed, comparing the Raman spectra in Figure 6 with the typical Raman spectra of different types of DLC displayed in Figure 33 of Ref. [60], the films synthesized in 0.5 and 1 mbar CH_4_ seem to belong to the a-C:H type of DLC. A further support for this hypothesis is that a-C:H density is ~2.4 g/cm^3^, identical to the density of the films bottom layer and much lower than the density of ta-C (~3.3 g/cm^3^) [60]. When ablated C atoms collide with CH_4_ molecules in the ambient, the number of possible emerging radicals can be quite high: CH_4_, CH_3_, CH_2_, CH, C, C_2_H_4_, C_2_H_3_, C_2_H_2_, C_2_H, C_2_, H_2_, and H [61]. In the case of 0.5 or 1 mbar CH_4_ ambients, even if initially the species reaching the substrate are sp^2^ or sp^1^ bonded, the presence of hydrogen species (broken from the methane molecules) in their vicinity will produce chemical reactions that result in the formation of C-C and C-H simple bonds (sp^3^ hybridization). In vacuum, because the carbon atoms are coming solely from the ablated target and there is no compensating hydrogen, there is a mix of sp^2^-sp^3^ bonds between the carbon atoms.

All films were made of two distinct layers with different thickness and density. The top layer had a lower density than graphite, while the bottom layer was significantly more compact (Table 2). The films synthesized in 0.5 and 1 mbar CH_4_ had a prominent top layer which surpassed 100 nm (~140 nm for the sample synthesized in 0.5 mbar CH_4_ and 150 nm for the sample synthesized in 1 mbar CH_4_). The density of the top layer was slightly higher in the case of the sample synthesized in 0.5 mbar CH_4_ (1.7 g/cm^3^ *vs.* 1.5 g/cm^3^), which might explain the higher values in hardness obtained for these films. The double stratification of PLD carbon films was assessed in Refs. [62,63] to “subplantation”, *i.e.*, implantation of ablated atoms and ions into the subsurface layer of the substrate. The continual bombardment of the substrate with carbon species causes the growth of a dense layer close to the surface. The highly energetic atoms in plasma will continue to penetrate this layer contributing to its densification, or stick on its surface, causing the increase of its thickness. On the other hand, the lower energy carbon species in plasma cannot penetrate this dense layer and remain on its surface, thus forming a lower density layer. Highly energetic species from subsequent pulses will be able to easily penetrate this soft layer and stick to the denser layer. The samples synthesized in vacuum had an extremely thin top layer (~30 nm) and a significantly thicker bottom layer (~290 nm), which might account for their drastically increased hardness values by 80%–85% as compared to the other samples, despite a lower sp^3^ content in the films (point black *vs* red in Figure 7). The thicker bottom layer of films deposited in vacuum *vs* CH_4_ ambient can be explained by the higher energy of species hitting the substrate, due to the lack of collisions with molecules of the ambient gas. However, this would mean that most PLD carbon films would be double structured, which is not the case. Budai *et al.* [61] synthesized carbon films in methane or hydrogen (pressures ranging between 10^−7^ and 0.5 mbar) using an ArF laser source, but observed no such double layering. In their case, the ambient influenced just the thickness of the films, the methane ambient favoring synthesis of 1.5 times thicker layers than in the case of hydrogen ambient. Modabber *et al.* [64] synthesized DLC films in vacuum, applying a constant number of pulses and changing only the substrate temperature between 100 and 300 °C. They observed that by increasing the substrate temperature, the ratio of sp^2^ bonding augmented and the mechanical properties decreased. However, no double layers were reported, and this is in agreement with our findings that in vacuum the soft top layer is extremely thin (few nm only). Growth of DLC films by PLD in inert gases, such as He and Ar, also produces monolayers [65]. In these cases the increase of the gas pressure caused a cooling down of the plume, which translated to in increase of sp^2^ content and clusterization of carbon atoms during flight. Taking into account the aforementioned examples, it seems that the double layering of films might be typical to reactive atmospheres. The fact that Budai *et al.* [61] did not obtain double layers, despite using a similar methane-based deposition ambient, might be due to the different laser wavelength employed in their experiments. K. Yamamoto *et al.* [66] studied the sp^3^ evolution function of laser wavelength for depositions conducted in vacuum, to exclude the background influence on films’ composition. They showed that sp^3^ content dropped from 80% when using 193 nm wavelength lasers (ArF) to about 40% when depositions were carried out with 248 nm wavelength (KrF). It is possible that the higher energy of the ablated carbon atoms allowed a higher mobility on the substrate and a better packing of the film.

## 5. Conclusions

Thin carbon films were synthesized by Pulsed Laser Deposition in vacuum or 0.5, 1, 10 mbar CH_4_ atmosphere on quartz and Si (100) substrates. The films had a very good compatibility with the Si (100) substrate, remaining adherent irrespective of the deposition temperature. For substrate temperatures below 200 °C, the films were not adherent to quartz, delaminating and forming flakes. At 200 °C, the films became adherent to quartz, but the bonding strength had values 40% lower compared to films synthesized on Si (100). The mismatch of the CTE was the most plausible cause for the layers incompatibility with the substrates. By introducing a buffer gradient layer with varying composition between substrate and films the bonding strength was increased by around 60%. The buffer had no effect on the bonding strength between Si (100) and the carbon films.

The deposition ambient played an important role in the films thickness. Increasing the CH_4_ content in the deposition chamber from 0 to 1 mbar increased the films’ thickness, due to plasma confinement which directed the ablated material towards the substrate. At 10 mbar CH_4_, the deposition rate was dramatically decreased by more than 95%, probably due to numerous collisions between ablated species and the CH_4_ molecules, resulting in a slowing down and even complete stop of the species on their track towards the substrate.

XPS and Raman analyses of films composition evidenced that layers synthesized in 0.5 or 1 mbar CH_4_ had the highest sp^3^ content, ~30% more than films synthesized in vacuum or 10 mbar CH_4_. However, their hardness was lower than 15 GPa and the elastic modulus indicated a plastic behavior. The samples with lower sp^3^ content were quite hard materials and possessed a higher elastic modulus compared to films synthesized in CH_4_ (200 *vs.* 75 GPa). Based on density and carbon hybridization ratio, the films synthesized in vacuum were assessed to be a-C type of DLC, while films synthesized in CH_4_ seem to belong to the a-C:H category.

The films synthesized in 0.5 and 1 mbar CH_4_ had higher transmittance compared to films deposited in vacuum. The transmittance increased towards higher wavelength range. The band gaps of films were in accordance with the sp^3^-sp^2^ content determined from Raman and XPS investigations, with lower values for samples with higher sp^2^ content.

XRR investigations evidenced that, in most cases, the PLD films were composed of two layers: one surface layer with density lower than graphite and a significantly denser layer underneath. The increased mechanical properties of samples with lower sp^3^ content were probably due to a decrease of the surface layer thickness down to negligible values.

## Supplementary Materials

Supplementary materials can be accessed at:  http://www.mdpi.com/1996-1944/8/6/3284/s1.
