# Supplementary material for: The Role of Ambient Gas and Pressure on the Structuring of Hard Diamond-Like Carbon Films Synthesized by Pulsed Laser Deposition
Source: Materials (Basel). 2015 Jun 5;8(6):3284–305. doi: 10.3390/ma8063284 (PMC5455729; doi:10.3390/ma8063284)
Supplement: Supplementary file 1 [file materials-08-03284-s001.pdf]

Supplemental Materials

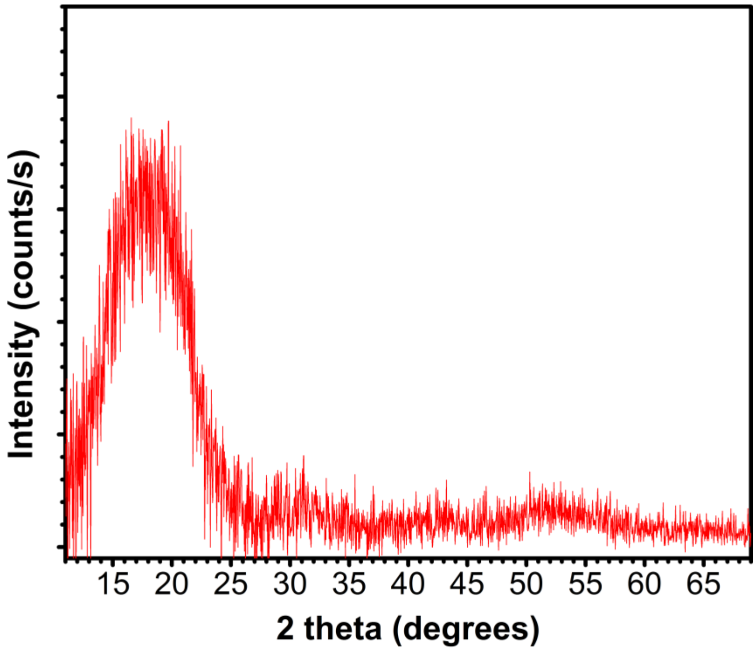

Figure S1. Typical GIXRD diffraction pattern of a carbon film synthesized at 200 °C by pulsed laser deposition (PLD) on a bare Si (100) substrate.
